# A Latent Class Approach for Classifying the Problem and Disordered Gamers in a Group of Adolescence

**DOI:** 10.3389/fpsyg.2018.02273

**Published:** 2018-11-26

**Authors:** Helga Myrseth, Guy Notelaers

**Affiliations:** Department of Psychosocial Science, University of Bergen, Bergen, Norway

**Keywords:** gaming, gaming disorder, problem gaming, assessment, gaming addiction scale for adolescents, latent class analysis

## Abstract

Gaming disorder is not yet recognized as a formal psychiatric disorder, and consensus is still lacking in the field concerning the definition of gaming disorder and what methods should be used to measure it. In order to deal with methodological challenges related to previously suggested approaches, the aim of the present study was to develop an alternative assessment procedure for gaming disorder using a latent class cluster approach, and to compare the criterion validity of this procedure with existing assessment procedures. A representative sample of 3,000 adolescents (*n* = 1,500 female) aged 17.5 years was drawn from the National Registry, and 2,055 participants responded (yielding a response rate of 70.3%). The Gaming Addiction Scale for Adolescents was used to measure gaming disorder and measures of loneliness, anxiety, depression, and aggression were used to test criterion validity. A model with five Latent Class Clusters represented the best fit [BIC(LL) = 21,253,7; L^2^ = 3,881,204; df = 1,978; Class. Err. = 0.1239]. The five different groups were labeled never symptoms (46.2%), rarely symptoms (22.3%), occasionally symptoms (23.5%), problem gamers (6.9%), and disordered gamers (1.2%). The groups displayed different probabilities of responses (never/rarely/sometimes/often/very often) to the seven Gaming Addiction Scale items. Regarding criterion validity, MANOVA revealed a significant overall main effect of latent classes [*F*_(20, 6359)_ = 13.50, *p* < 0.001; Wilks Lambda = 0.871]. All dependent variables (loneliness, depression, anxiety, verbal, and physical aggression) reached statistical significance when results from the dependent variables were considered separately. Comparing the present approach with previous suggested classifications of gaming addiction offered by Lemmens et al. and Charlton and Danforth, the present approach showed greater specificity in terms of number of classes identified. We conclude that the Latent Class approach identifying five different groups of gamers offers a more refined view on addiction compared to previous assessment procedures.

## Introduction

The majority of adolescents plays electronic games regularly, but for some individuals the gaming becomes excessive and leads to functional impairment and psychological distress. Several different terms have been used to describe the state where gaming becomes problematic, e.g., gaming addiction, problem gaming, pathological gaming, compulsive gaming, and gaming disorder. Consensus is still lacking concerning the definition of gaming disorder, which terms to use, and what methods should be used to measure it (Brunborg et al., 2014). Although gaming disorder is not yet recognized as a formal psychiatric disorder, the term “Internet Gaming disorder” has been included in the “Emerging measures Models” section of the latest version of the Diagnostic and Statistical Manual of Mental Disorders−5 (American Psychological Association, 2013) and the beta draft of the 11th revision of the International Classification of Diseases (ICD-11) includes “Gaming disorder” in the section “Disorders due to Substance Use or Addictive Behaviors” (World Health Organization, 2017). The term *gaming disorder* will be used in the following when referring to the state of “excessive and compulsive use of computer or video games that results in social and/or emotional problems; despite these problems, the gamer is unable to control this excessive use” (p. 78, Lemmens et al., 2009).

Prevalence rates of gaming disorder has been estimated to be between 2 and 8.5% (Gentile, 2009; Lemmens et al., 2009; Wenzel et al., 2009; Rehbein et al., 2010), where younger males are heavily overrepresented (Lemmens et al., 2009; Rehbein et al., 2010). Gaming disorder has been linked to increased aggressive behavior, lower physical, and psychological well-being, lower productivity, and impoverished relationships (Anderson and Bushman, 2001; Mathers et al., 2009; Brunborg et al., 2011; Mentzoni et al., 2011), but these negative outcomes are not necessarily related to time spent gaming (Brunborg et al., 2014).

The classification of gaming disorder has much relied on diagnostic approaches for other addictions, e.g., gambling disorder or substance addiction, which may result in misclassifying highly engaged gamers as having a disorder (Carras and Kardefelt-Winther, 2018). There has been two basic approaches in the field to diagnosing disordered gaming (Ferguson et al., 2011). The first approach is inspired by the DSM criteria in which a certain number of criteria must be endorsed in order to be classified as a disordered gamer, whereas the other approach recognizes that the criteria have different specificity and that some criteria may measure engagement rather than addiction. A meta-analysis of gaming disorder found that studies using the first approach tended to overestimate the prevalence because individuals who might be better classified as highly engaged were categorized as problem/addicted gamers (Ferguson et al., 2011).

Lemmens et al. (2009) developed the Gaming Addiction Scale for Adolescents (GASA). based upon the six suggested criteria for behavioral addiction (Brown, 1993; Griffiths, 2005; Griffiths and Davies, 2005): Salience (preoccupation with gaming), tolerance (increasing engagement), mood modification (gaming to relieve aversive emotional states), withdrawal (psychological/physical discomfort when gaming is reduced/discontinued), relapse (unsuccessful attempts to cut down/stop gaming), and conflict (intra/interpersonal conflicts resulting from excessive gaming). In addition to these six criteria, they included an item measuring problems caused by excessive gaming (neglecting/putting off other activities e.g., social engagement/work tasks). For each item, respondents indicate on a 5-point scale how often each incident has occurred during the last 6 months. A score of 3 (sometimes) or above indicates that the criterion is endorsed. Lemmens suggested that endorsing four or more criteria indicates problem gaming whereas all seven criteria must be endorsed in order to be classified as an addicted gamer.

Charlton (2002) is an advocate of the other approach to diagnosing gaming addiction. He suggested that engagement may be viewed as a positive and non-interfering experience, and proposed that high engagement (with the absence of withdrawal symptoms) should be distinguished from addiction. Two factors were identified; one addiction factor tapping items suggested to measure the core addiction criteria (conflict, withdrawal, relapse/reinstatement, and behavior salience) and one engagement factor tapping items measuring peripheral addiction criteria (cognitive salience, tolerance, and euphoria) (Charlton, 2002; Charlton and Danforth, 2007). Charlton suggested that either the core criteria alone should be used when classifying addiction, or the core criteria should be given greater weighting in the classification of problem gaming. The existence of a developmental process was suggested based on analysis of response frequencies where peripheral criteria are met before the core criteria (Charlton and Danforth, 2007). Recently, several studies have applied Charlton and Danforth's classification on the GASA instrument in order to establish prevalence of problem gaming and gaming addiction (e.g., Brunborg et al., 2013; Wittek et al., 2016).

However, both of these approaches are problematic for three reasons. First, they both use a Likert-type scale which is subsequently dichotomized into endorsing the criteria or not. Hence, one assumes that a score of 3 (sometimes) on the GASA is equal to a score of 5 (very often) which conceptually may be problematic because the difference between endorsing a symptom never and rarely can elicit important information as well as the difference between endorsing a symptom sometimes, often, and very often. Second, Lemmens' approach relies on using cut-off points to distinguish between addicted and non-addicted gamers, which are rather arbitrary. Third, by making a sum score this approach assumes–irrespective of the call for using different weights–that all indicators have equal properties; i.e., item difficulty, item popularity, and discriminatory power. Finally, if one were to assume an interval measure, one cannot assume that the GASA items do not follow a normal distribution. In fact, for the three last items the skewness varied between 2 and 3 whereas the kurtosis varied between 3.8 and 7.8. Such a skewness and kurtosis points clearly to a violation of the assumption of normality that underlies classical measurement models like the principal components and the factorial. Using such method would hamper the statistical validity as it would result in untrustworthy parameter estimates (Trochim, 2000; Vermunt and Magidson, 2005).

Like Carras and Kardefelt-Winther (2018) and Deleuze et al. (2017), we propose an alternative procedure to classifying respondents into different gaming groups and aim to investigate its criterion validity in comparison to both existing procedures. To deal with the four challenges we propose a latent class analysis (LCA) approach as an alternative strategy for identifying the different groups of gamers (Deleuze et al., 2017; Carras and Kardefelt-Winther, 2018). First, in contrast to more classical cluster methods, LCAcan easily treat categorical response variables as they are used in gaming (Magidson and Vermunt, 2002a): “never,” “rarely,” “sometimes,” “often,” and “very often.” Secondly, LCA is a rather non-arbitrary way to identify subgroups because it uses statistical criteria to decide the appropriate number of types of gamers i.e., the Bayesian Information Criterion and the Bootstrapping procedure of the Log-likelihood (see section on Model Selection). This may have important implications for establishing more accurate prevalence rates of gaming addiction. We expect that applying LCA approach will identify different groups that differ in severity of gaming problems, and we also aim to validate our findings in terms of criterion validity. Based on previous findings, we expect more severe gaming problems to be related to higher levels of loneliness (Kim et al., 2009; Lemmens et al., 2011), depression (Allison et al., 2006; Mentzoni et al., 2011), anxiety (Allison et al., 2006; Mentzoni et al., 2011), and aggression (Anderson and Bushman, 2001). Third, LCA takes item properties such as item popularity and discriminatory power into account (Vermunt, 2001). Finally, LCA does not rely on strong distributional assumptions like normality. In LCA, the latent variable is assumed to be discrete and to come from a multinomial distribution (Vermunt and Magidson, 2005).

A recent study in the field utilized an LCA to identify gaming disorder and found that using the DSM-5 based approach tended to misclassify almost 1/3 of their sample (Carras and Kardefelt-Winther, 2018); 7.3% were misclassified as disordered gamers while only engaged gamers and 23.6% reported moderate to high levels of problems but few symptoms and failed to be identified as potential problem gamers. The endorsement of symptom criteria (e.g., preoccupation, tolerance, loss of control, and withdrawal) corresponded with the reporting of problem criteria related to gaming (e.g., difficulty in school or with family) in only two of the five groups they identified.

## Materials and methods

### Design and setting

A sample of adolescents was randomly drawn from the Norwegian National Registry, and received a postal invitation to participate in a survey about gambling and gaming. The questionnaire could be completed online or on paper (returned in a pre-paid return envelope). Two reminders were sent to those who did not respond. All respondents received a gift certificate worth NOK 200 (~24 EUR) for participating.

### Participants

This study was a part of a larger longitudinal study investigating prevalence, correlates, and patterns of change between mental health symptoms and different addictions (chemical and behavioral) in the transition from adolescence to young adulthood. Three thousand adolescents (50% female) aged 17.5 years were randomly sampled to participate in the first wave. A small minority of individuals (*n* = 77) were excluded from the study due to invalid mailing addresses or because they were unable to participate (e.g., due to disability). Of the remaining sample, 2055 participants completed and returned the questionnaire, resulting in a response rate of 70.4%. Approximately half of the respondents were female (52.9%)^1^. The majority was born in Norway (92.4%), and most respondents went to school full time (97.7%).

### Measures

#### Disordered gaming

The Gaming Addiction Scale–Adolescents (GASA) (Lemmens et al., 2009) includes seven items measuring salience, tolerance, mood modification, withdrawal, relapse, conflict, and problems caused by gaming. Respondents indicate on a scale from 1 to 5 how often each problematic incident has occurred during the past 6 months (never, almost never, sometimes, often, very often). Cronbach's Alpha for the GASA was 0.88.

#### Loneliness

Loneliness has been found to predict subsequent gaming addiction (Seay and Kraut, 2007; Lemmens et al., 2011) and was hence included as a measure of criterion validity. Loneliness was measured using Robert's UCLA Loneliness Scale (Roberts et al., 1993), consisting of eight items, with the following response categories: never, seldom, sometimes, and often. The scoring of four items is reversed so that higher scores are indicative of higher levels of loneliness. Cronbach Alpha was 0.76.

#### Depression and anxiety

Depression and anxiety are often correlated with loneliness (Myrseth et al., 2017) and elevated levels of depression and anxiety have been found among individuals with gaming problems in numerous studies (Allison et al., 2006; Seo et al., 2009; Mehroof and Griffiths, 2010; Mentzoni et al., 2011). The Hospital Anxiety and Depression Scale (HADS) (Zigmond and Snaith, 1983) was therefore included in the present study, and is a 14-item instrument measuring severity of anxiety and depression. The HADS has been validated in different populations, and is short and sensitive (Herrman, 1997). The seven items in each subscale have a scoring range of 0–21 where higher scores indicate more severe symptomatology. Cronbach Alphas were 0.76 and 0.69, for the anxiety and depression subscales, respectively.

#### Aggression

Aggression has also been found to be a significant predictor of gaming addiction (Mehroof and Griffiths, 2010), and was therefore included as a criterion variable in the present study. Aggression was measured using the *Buss-Perry Aggression Questionnaire Short-Form* (BPAQ-SF) (Diamond and Magaletta, 2006). Two of the four subscales (physical aggression and verbal aggression) were included in the present study. Each item is rated on a 5-point Likert scale ranging from 1 (very unlike me) to 5 (very like me). Cronbach Alphas of 0.80 and 0.66 were found for the subscales physical aggression and verbal aggression, respectively.

### Ethics

The study was conducted in accordance with the Declaration of Helsinki, and written consent was given by all participants. As all respondents in the present study were above 16 years of age, parental participation consent was not necessary according to Norwegian legislation. The project was approved by the Regional Ethics Committee, Health Region South East of Norway (project number 2012/914).

### Statistical analyses

Latent Class Analysis was used to determine disordered gaming, an analysis which is very suited because it does not dependent upon distributional assumptions. LCA (Magidson and Vermunt, 2001, 2002b, 2004; Vermunt, 2001; Vermunt and Magidson, 2002;) is a statistical method that systematically classifies respondents into mutually exclusive groups with respect to a given trait (gaming addiction) that is not directly observed (manifest). The classes are not directly observable, they are latent (Vermunt, 2004). One empirically investigates whether the assumption about the relationship between the latent variable (e.g., addiction) and the frequencies of reported behaviors (e.g., gaming) is acceptable. LCA enables us to identify mutually exclusive groups that adequately describe the dispersion of observations in the n-way contingency table of discrete variables (i.e., gaming behaviors). The goal of LCA is to determine the smallest number of latent classes, sufficiently explaining the associations observed between the manifest variables (GASA items) (Magidson and Vermunt, 2004).

The starting point for a latent class model is homogeneity, i.e., everybody resides in the same group. This baseline model is a 1-latent class (LC) cluster model. In a LC cluster model, clusters are subsequently added. A n-cluster model may result in latent classes that differ in function of the nature and the frequency of reported symptoms. The metric of this single latent variable is typically nominal. Instead of increasing the number of clusters, the number of latent variables (factors) may be increased as well. Magidson and Vermunt (2001) labeled this type of latent class models as LC Factor models because of the natural analogy to standard factor analysis. Because it has been theorized that gaming addiction may be consisting of two factors (core and peripheral symptoms/criteria) we will compare a 2-factor solution with a cluster solution. Like with traditional confirmatory factor models, a priori knowledge about the relationship between items and latent variables is needed (Vermunt and Magidson, 2005). Moreover, with traditional measurement models, the (discrete) latent variable must adequately explain the initial relationship between the indicators, i.e., symptoms.

For estimating latent classes of disordered gaming we used the software package Latent Gold 5.1 (Vermunt and Magidson, 2016). Evaluating the fit of LC models is not straightforward. There are many indicators of fit and rules of thumb that should be taken into account (Reknes et al., 2017). The Bayesian Information Criterion (BIC) is most often used for model selection. Mccutcheon (1987) and Hagenaars (1990) suggested to accept the model with the lowest BIC because the models are non-nested. For model fit, the χ^2^ is bootstrapped (Langeheine et al., 1996). In addition to statistical fit measures, it is also important to inspect local fit and the quality of the classification. To inspect the origins of misfit, i.e., local fit, we used the bivariate residuals (BVRs). The BVRs show how much association between each pair of indicators remains in the measurement model, using the 1-cluster model as a reference. Ideally, the value should be lower than 3.84, a value corresponding to a significant χ^2^ with 1 degree of freedom (Statistical Innovatations, 2013). However, as the *L*^2^ follows a χ^2^ distribution, the BVRs are also quite sensitive for large sizes. Therefore, we suggest using a more relative threshold, where the reduction of the BVRs should be at least 90% (Notelaers et al., 2006). Finally, we also assessed how well the classes are separated by inspecting the total rate of classification errors due to adjacent erroneous classification.

In order to simultaneously test the external validity (i.e., whether the latent classes scored significantly different on loneliness, depression, anxiety, verbal, and physical aggression) the latent class classifications were exported to a SPSS file (De Cuyper et al., 2008), where Multivariate analysis of variance (MANOVA) analysis was conducted. MANOVAs were also conducted separately for the Lemmens' and for the Charlton and Danforth's classifications.

## Results

### Model selection

Table 1 gives an overview of the different measurement models estimated with Latent Gold-5 and respective fit measures showing the iterative procedure associated with selecting the most appropriate LC model.

**Table 1:** Fit statistics of the Latent Class models.

**Models**	**BIC(LL)**	***L*2**	**df**	**Class.Err**.
1. Base -LC 1 cluster model	27198, 9	10077, 87	2011	0
2. LC 5 clusters model	21407, 92	4043, 037	1979	0.1008
3. LC 6 clusters model	21402, 32	3976, 475	1971	0.1087
4. LC 7 clusters model	21390, 37	3903, 567	1963	0.118
5. LC 8 clusters model	21418, 23	3870, 463	1955	0.174
6. Latent Class factor model. 2 Factors with each 4 latent classes: GAS-1 and GAS-2 as single factor related to the other factor comprising of other GAS items (4,4)	21380, 74	4137, 78	1995	0.2683
7. Latent Class factor model. 2 Factors with each 4 latent classes: GAS-1 and GAS-2 as single factor unrelated to the other factor comprising of other GAS items (4,4)	21378, 45	4143, 114	1996	0.2626
8. LC 1 cluster model GAS-1 and GAS-2 correlated	25807, 84	8679, 191	2010	0
9. LC 2 clusters model, GAS-1 and GAS-2 correlated	22079, 24	4889, 625	2002	0.0267
10. LC 3 clusters model, GAS-1 and GAS-2 correlated	21402, 2	4151, 623	1994	0.0574
11. LC 4 clusters model, GAS-1 and GAS-2 correlated	21258, 16	3946, 621	1986	0.0984
***12***. LC 5 clusters model***, GAS-1 and GAS-2 correlated***	***21253, 7***	***3881, 204***	***1978***	***0.1239***
13. LC 6 clusters model, GAS-1 and GAS-2 correlated	21285, 91	3852, 452	1970	0.1267
14. LC 7 clusters model, GAS-1 and GAS-2 correlated	21315, 37	3820, 951	1962	0.1252

*LC, Latent Class; BIC, Bayesian Information Criterion; LL, Log Likelihood; L^2^, squared log-likelihood; Class. Err., Classification Errors. The model with the best fit is presented in bold and italic*.

The first model with the lowest BIC is the 7-LC cluster model, which also statistically fits since the bootstrap of the *L*^2^ is non-significant. Yet, inspection of local fit with the BVRs yielded that the residual of the first and second items were relatively high. Further inspection of residuals of the 4-, 5-, and 6-LCA models pointed toward a pitfall Uebersax (2009) has warned about; when a BVR is particularly high, additional clusters may emerge primarily based upon the symptoms in questions. This is suboptimal and can be circumvented by adding a latent variable that accounts for the residuals (model 6 and 7) or allowing the residuals to correlate (model 8). In model 6 and 7 a second latent variable was added. This is in line with Charlton and Danforth (2007) who advocated that there is a difference between engaged and addicted gaming. The difference between model 6 and 7 is that the engaged gaming factor is related to the addiction factor in model 6 whereas in model 7 both factors are unrelated. From model 8–14 we estimated a single latent variable model allowing for an error-correlation between item 1 and 2. These models reveal that engaged gaming may be a result of shared variance between two items that is unrelated to the addiction typology. The BIC yielded that adding a latent variable is more adequate than adding clusters (model 1–5). Because the BIC of model 7 is lower compared to model 6 we may conclude that both items measure something different from and unrelated to the rest of the GASA items. Yet, the BIC of model 12 is even lower. Hence, allowing for the residuals of item 1 and 2 to correlate while estimating a 5 cluster LC GASA model is most adequate. The bootstrap *p*-value of the *L*^2^ of this model is 0.15 which is higher than 0.05. Hence, this model is not only the most appropriate measurement model, but also fits well.

### Meaning of the classes

Conditional probabilities allowed us to depict the precise meaning of the latent classes, see Table 2. The sizes of the different classes for each factor are presented as percentages on the first row.

**Table 2:** Conditional Probabilities of responses to the seven GASA indicators.

**GASA indicators**	**Response scales**	**Distribution of the five latent classes**				
		**Never**	**Rarely**	**Occasionally**	**Problem**	**Disordered**
		**(46.2%)**	**(22.3%)**	**(23.5%)**	**(6.9%)**	**(1.2%)**
GASA-1: Did you think about playing a game all day long?	Never	0.9694	0.4872	0.2217	0.0569	0.0004
	Rarely	0.0303	0.3580	0.3541	0.1936	0.0075
	Sometimes	0.0004	0.1315	0.2926	0.3455	0.0683
	Often	0	0.0213	0.1084	0.2776	0.2778
	Very often	0	0.0020	0.0232	0.1264	0.6459
GASA-2: Did you spend increasing amounts of time on games?	Never	0.9748	0.3222	0.1026	0.0201	0.0021
	Rarely	0.0250	0.4453	0.3351	0.1405	0.0364
	Sometimes	0.0002	0.2020	0.3880	0.3712	0.2345
	Often	0	0.0285	0.1465	0.3272	0.4294
	Very often	0	0.0020	0.0278	0.1410	0.2975
GASA-3: Did you play games to forget about real life?	Never	0.9663	0.5143	0.2996	0.0947	0.0042
	Rarely	0.0321	0.2484	0.2408	0.1441	0.0189
	Sometimes	0.0016	0.1797	0.2901	0.3285	0.1288
	Often	0	0.0495	0.1331	0.2851	0.3333
	Very often	0	0.0081	0.0364	0.1477	0.5147
GASA-4: Have others unsuccessfully tried to reduce your game use?	Never	0.9957	0.6534	0.2278	0.0249	0
	Rarely	0.0043	0.2685	0.3398	0.1241	0.0014
	Sometimes	0	0.0722	0.3317	0.4045	0.0436
	Often	0	0.0058	0.0968	0.3941	0.4153
	Very often	0	0.0001	0.0039	0.0524	0.5397
GASA-5: Have you felt bad when you were unable to play?	Never	0.9977	0.9669	0.5937	0.1791	0.0010
	Rarely	0.0023	0.0325	0.3160	0.3552	0.0174
	Sometimes	0	0.0005	0.0838	0.3512	0.1459
	Often	0	0	0.0063	0.0982	0.3466
	Very often	0	0	0.0003	0.0163	0.4891
GASA-6: Did you have fights with others (e.g., family, friends) over your time spent on games?	Never	0.9959	0.9594	0.5347	0.1268	0.0253
	Rarely	0.0041	0.0397	0.3342	0.2902	0.1232
	Sometimes	0	0.0009	0.1156	0.3673	0.3319
	Often	0	0.0144	0	0.1674	0.3220
	Very often	0	0.0011	0	0.0483	0.1977
GASA-7: Have you neglected other important activities (e.g., school, work, sports) to play games?	Never	0.994	0.4199	0.7572	0.1419	0.0112
	Rarely	0.006	0.3180	0.1962	0.2418	0.0540
	Sometimes	0	0.2026	0.0428	0.3466	0.2185
	Often	0	0.0511	0.0037	0.1968	0.3503
	Very often	0	0.0084	0.0002	0.0728	0.3661

**Figure 1:**
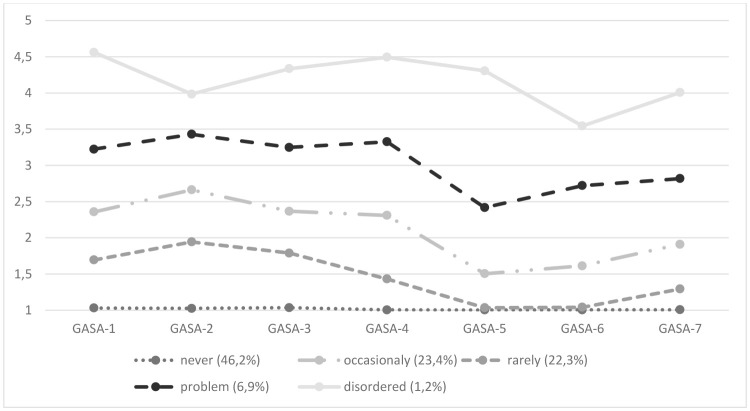
Latent Class Profile Plot of average scores on the GASA indicators.

We chose to simplify this table by also portraying the conditional means in Figure 1,^2^. These means are the average score to an item given the latent class membership^3^.

Forty-six percent of the sample belonged to the first class labeled ‘never symptoms of addiction' as the average response to the GASA items was 1 (never). The second class, constituting 22%, had average responses to the GASA items between 1 and 2 (never and rarely). We suggest labeling them ‘rarely symptoms of addiction'. The third class of respondents (23%) occasionally showed symptoms of addiction. Their average probability to respond rarely or more often to the first four GASA items was above 0.50, whereas the probability to respond never to item 5 and 6 of GASA was over 0.95. Almost 7% of the sample showed often symptoms of addiction with average probability of 0.80 to respond sometimes or more frequent to the GASA items, and was labeled “problem gamers.” The smallest latent class (1.2%) showed very often symptoms of addiction with an average probability of almost 0.80 to endorse 4 or 5 (often/very often) and were labeled “disordered gamers.”

### Criterion validity

The MANOVA revealed a significant overall main effect of latent classes [*F*_(20, 6359)_ = 13.50, *p* < 0.001; Wilks Lambda = 0.871]. When the results from the dependent variables were considered separately, all dependent variables reached statistical significance, see Table 3.

**Table 3:** Multivariate analysis of variance (MANOVA) for differences between the five latent clusters: Never (*n* = 952), Rarely (*n* = 460), Occasionally (*n* = 482), Problem (*n* = 142), and Disordered (*n* = 24).

	**LCA classification (LCA)**					**Charlton and danforth's classification (CD)**						**Lemmens' classification (L)**					
			**Between subject differences**						**Between subject differences**						**Between subject differences**		
***Variables***	**Z Mean**	**SD**	***F***	**Sig**	**η^2^**	**Variables**	**Z Mean**	**SD**	***F***	**Sig**	**η^2^**	**Variables**	**Z mean**	**SD**	***F***	**Sig**	**η^2^**
**Loneliness**			22.15	0.000	0.044	**Loneliness**			20.61	0.000	0.031	**Loneliness**			31.87	0.000	0.032
Never^c^, ^d^, ^e^	−0.13	0.92															
Rarell	−0.10	0.92				f. Non-problem^g^, ^h^, ^i^	−0.08	0.95				j. Non-problem^k^, ^l^	−0.06	0.95			
Occasionalll^a^, ^b^, ^d^, ^e^	0.16	1.06				g. Engaged^f^	0.30	1.17									
Problem^a^, ^b^, ^c^	0.58	1.20				h. Problem^f^	0.36	1.12				k. Problem^j^, ^l^	0.40	1.15			
Disordered^a^, ^b^, ^c^	0.89	1.38				i. Addicted^f^	0.66	1.23				l. Addicted^j^, ^k^	0.88	1.37			
**Depression**			24.99	0.000	0.049	**Depression**			23.58	0.000	0.035	**Depression**			39.77	0.000	0.040
Never^c^, ^d^, ^e^	−0.15	0.94															
Rarely^c^, ^d^, ^e^	−0.11	0.95				f. Non-problem^g^, ^h^, ^i^	−0.08	0.96				j. Non-problem^k^, ^l^	−0.08	0.96			
Occasionally^a^, ^b^, ^d^, ^e^	0.22	1.01				g. Engaged^f^, ^i^	0.39	1.15									
Problem^a^, ^b^, ^c^	0.55	1.17				h. Problem^f^, ^i^	0.33	1.09				k. Problem^j^, ^l^	0.46	1.13			
Disordered^a^, ^b^, ^c^	0.73	1.22				i. Addicted^f^, ^g^, ^h^	0.76	1.17				l. Addicted^j^, ^k^	0.94	0.97			
**Anxiety**			2.99	0.018	0.006	**Anxiety**			3.09	0.026	0.005	**Anxiety**			3.22	0.040	0.003
Never^d^	−0.01	1.01															
Rarely^c^, ^d^	−0.11	0.98				f. Non-problem^g^, ^i^	−0.03	1.00				j. Non-problem	−0.03	1.00			
Occasionally^b^	0.02	0.97				g. Engaged^f^	0.22	2.01									
Problem^a^, ^b^	0.22	1.03				h. Problem	0.03	1.00				k. Problem	0.10	0.99			
Disordered	0.20	1.24				i. Addicted^f^	0.28	1.09				l. Addicted	0.34	1.07			
**Verbal Aggression**			21.50	0.000	0.043	**Verbal Aggression**			15.32	0.000	0.023	**Verbal Aggression**			23.32	0.000	0.024
Never^b^, ^c^, ^d^, ^e^	−0.19	0.96															
Rarely^a^, ^c^, ^d^, ^e^	−0.01	0.87				f. Non-problem^h^, ^i^	−0.07	0.96				j. Non-problem^k^, ^l^	−0.06	0.96			
Occasionally^a^, ^b^, ^d^	0.21	1.04				g. Engaged^h^, ^i^	0.05	1.03									
Problem^a^, ^b^	0.46	1.11				h. Problem^f^, ^g^	0.35	1.10				k. Problem^j^	0.35	1.12			
Disordered^a^, ^b^	0.60	1.27				i. Addicted^f^, ^g^	0.56	1.25				l.Addicted^j^	0.71	1.31			
**Physical Aggression**			37.78	0.000	0.073	**Physical Aggression**			32.52	0.000	0.048	**Physical Aggression**			50.31	0.000	0.050
Never^b^, ^c^, ^d^, ^e^	−0.21	0.89															
Rarell^a^, ^c^, ^d^, ^e^	−0.04	0.93				f. Non-problem^g^, ^h^, ^i^	−0.10	0.92				j. Non-problem^k^, ^l^	−0.09	0.94			
Occasionally^a^, ^b^, ^d^, ^e^	0.18	1.02				g. Engaged^f^, ^h^, ^i^	0.20	0.99									
Problem^a^, ^b^, ^c^	0.70	1.26				h. Problem^f^, ^g^, ^i^	0.47	1.25				k. Problem^j^, ^l^	0.47	1.19			
Disordered^a^, ^b^, ^c^	1.13	1.34				i. Addicted^f^, ^g^, ^h^	0.86	1.33				l. Addicted^j^, ^k^	1.17	1.00			

post-hoc comparisons are indicated by listing the superscript letters of the groups from which they differ significantly at p < 0.05

a Never (LCA);

b Rarely (LCA);

c Occasionally (LCA);

d Problem (LCA); and

e Disordered (LCA);

f Non-problem (CD);

g Engaged (CD);

h Problem (CD);

i Addicted (CD);

j Non-problem (L);

k Problem (L);

l *Addicted (L)*.

*post-hoc* tests revealed that the problem and disordered groups scored significantly higher than the other groups on loneliness (*p* < 0.001), depression (*p* < 0.05–0.001), and physical aggression (*p* < 0.001); and significantly higher than the never symptoms (*p* < 0.001) and rarely symptoms (*p* < 0.01) groups on verbal aggression. The problem group scored also significantly higher than the never and rarely symptoms groups on anxiety. The never and rarely symptoms groups scored significantly lower than the other groups on most variables, see Table 3 for more details.

We compared the criterion validity of the LCA method with two existing cut-off methods (Charlton and Danforth, 2007; Lemmens et al., 2009) on the external validation criteria. To facilitate comparison we standardized the scales. Given the number of comparisons we summarize only main findings (details are presented in Table 3). Investigation of Lemmens' traditional cut-off points clearly shows that the dominant pattern is non-problem < problem < addicted group on the external criteria except from anxiety were the groups do not differ. When comparing eta^2^ of the Lemmens' classification with the Charlton &Danforth's (CD) classification we hardly see any difference. The main difference between these classifications is that the CD classification included an engaged gamer group. Hence, identifying an engaged gamer group does not influence the effect size. The pairwise comparisons show that the differences between the gamer groups in the CD classification are less clear compared to the Lemmens' classification. The engaged gamer group has similar scores as the problem group or the non-problem group, depending upon the criterion. Hence, the status of the engaged gamers seems rather ambiguous. Furthermore, the CD addicted group does not differ from the CD problem group on three of the external validation criteria; and significant differences between the engaged, problem, and addicted groups are absent for loneliness and anxiety. Finally, the z-scores of the Lemmens' addicted group are more negative than the CD addicted group. When comparing eta^2^ of the Lemmens and CD classification with the LCA classification we note a slight increase, indicating larger effect sizes of the LCA. The dominant pattern of the differences in the LCA is that never symptoms = rarely symptoms < occasionally symptoms < problem gamers = disordered gamers. Thus, with respect to the external criteria, the occasional symptoms group are different from the rarely symptoms and from the problem gamers.

## Discussion

The purpose of the present study was to develop a more accurate assessment procedure of gaming disorder, and results showed that a model with five latent class clusters represented the best fit. According to this model, there are five separate groups with different probabilities to choose the different responses to the seven GASA-items. There were two problematic groups (disordered and problem gamers), one middle group (occasional symptoms gamers), and two non-problematic groups (rarely and never symptoms gamers). The largest group (46%) was the never symptom group with 97% or higher probability of answering never to all GASA-items, reflecting that they in practice are non-gamers. The rarely symptoms group, comprising 22%, had a probability above 92% for answering never/rarely to the core addiction items (measuring relapse, withdrawal, and conflict) and 76–85% probability of answering never/rarely to the peripheral addiction criteria. Almost 7% of the sample belonged to the problem group which had the highest probability of scoring sometimes/often to most of the GASA-items. The disordered gamers, constituting 1.2%, had a high probability (above 80%) of scoring often/very often to most of the GASA-items.

The results further supported the external validation of the five latent class clusters. The problem and disordered group scored higher than the other groups on all measures except for anxiety. For anxiety the results were less clear, but the problem group scored significantly higher than the never and rarely symptoms groups; and the occasionally symptoms group scored significantly higher than the rarely symptoms group. Taken together, these results further support the external validity of differentiating between the five latent class clusters.

Recently, Pontes et al. (2014) also used a latent class model to distinguish between different groups of online gamers and they also arrived at five different clusters. The results of the present study were quite similar to that of Pontes et al. (2014), yet, as the present study used a representative sample we also found a group that did not game at all. In contrast to Pontes et al. (2014), we did not report a low risk high engagement class. This difference may be due to different types of samples and measurement instruments in the two studies. Nevertheless, in the present study, a class of engaged gamers came to the fore (model 3–5, table z) when distinguishing more than 5 classes. However, following Uebersax (2009) and his recommendations to also closely inspect the bivariate residuals showed that the engagement class was possible due to a high bivariate residual between the first and the second item of the GASA (the items that characterize the engaged class). Allowing this bivariate residual to correlate (model 12) resulted in an improved fit. Hence, it is possible that the engaged class is a statistical artifact which researchers using LCA should check closely when distinguishing between different types of gamers.

Comparison of the LCA method in the present study with the previous suggested classifications of gaming addiction offered by Charlton and Danforth (2007) and Lemmens et al. (2009), showed that the LCA approach is more specific compared to the other classifications of problem and addicted gamers. Although the z-scores of problem gamers on the external validity criteria are lower than those of addicted gamers in the LC model, they are not significantly different. Hence, it may seem like the LCA only adds additional group(s) without improving the previous classifications. However, a problem with the Lemmens' and CD classifications is that they do not differentiate between people who game regularly (without gaming problems) and those who (almost) never game. Furthermore, the mean z-scores on the external criteria of the LCA disordered group are quite similar to those of the Lemmens' addicted group while the means z-scores of the LCA problem group are similar to those of the CD addicted group. Hence, the LCA approach seems to reconcile or integrate both classifications, offering a more refined view on addiction.

Compared to the Lemmens' classification the LCA also distinguished rarely and occasional symptoms gamers. Although the rarely symptoms gamers are almost indistinguishable from the non-gamers with respect to the external criteria, they add to the description of the reality of gaming. In addition to people who never game, we identified another group who apparently participate in gaming but very rarely think about gaming all day long, very rarely spends increasing amounts of time gaming, hardly play games to forget about reality, very rarely neglect other activities due to gaming, hardly have unsuccessful attempts to reduce gaming, never have fights with others about time spent gaming and never feel bad when they cannot play. There is also a group who occasionally reports the first four addiction criteria but very rarely feels bad when they are unable to play, very rarely reports fighting with others, and very rarely neglects other activities due to gaming. With respect to the external criteria, this group is significantly different from all other groups. Although they occasionally endorsed the items suggested to measure engagement; we would not advise labeling this group as engaged. Frequency of reported addiction criteria may not qualify as engagement which is often defined as a state where a persons is highly dedicated, vigorous and absorbed by an activity (Schaufeli et al., 2002).

Although Lemmens' classification seem to have the highest external validity in our study, the question arises whether these three categories coincides well with how addiction develops which may have ramifications for developing interventions for gamers. From a developmental perspective, Lemmens' categorization implies that a gamer transitions immediately from a non-problem state to a problem or addicted state. Particularly problematic is the Lemmens' non-problem group which seems to include three different groups, i.e., the never, rarely and occasionally symptoms groups. Not recognizing the possible existence of different non-problem groups implies assuming that all subjects in the non-problem group have similar probability to transition into the other symptom groups of addiction. The conditional probabilities and the criterion of validity seem to suggest that this is not the case. In particular, the never group may have a very small probability to transition into problem/disordered group. The probability of the occasional symptom group to transition into problem gaming may also be different from the rarely symptoms group. For most of the external criteria, the distance between the occasional group and the rarely group is equal to the distance between the occasional and the problem group. If future research aims to describe and explain the development of gaming addiction, it seems rather strange to assume that the transition probabilities to later stages of addiction are the same for all of these three groups. The LC markov model allows for future research to empirically test whether these probabilities are different, which may have important implications for developing and applying primary and secondary interventions for the different groups of gamers.

### Practical implications

As the latent class cluster approach offers a more accurate assessment and categorization of different types of gamers, treatment of gamers may be enhanced by allowing for the implementation of targeted treatment interventions for the different types of gamers (e.g., prevention strategies for the occasional group, minimal interventions or self-help groups for the problem group, and treatment for the disordered group).

From a primary and secondary prevention point we suggest that future research should aim to estimate the transition probabilities that different types of gamers will switch between classes over time. In addition, we suggest that future research should investigate escalating and de-escalating predictors of these transition probabilities to prevent gamers to switch to a more problematic gamer group and to promote a switch from a problematic gaming class to a lesser or non-problematic gaming class.

### Limitations and future research

One important limitation is common method bias which may partially account for the strength of the relationship between GASA and the criterion variables. To circumvent common method variance, future research may collect data from registries and clinical records to establish the criterion validity.

Another limitation, of the present as well as previous studies, is that sensitivity and specificity could not be tested due to the lack of a golden standard. Estimation of a Receiver Operation Curve (Zweig and Campbell, 1993; Streiner and Cairney, 2007) could help to establish cut-off scores with the highest sensitivity and specificity. However, discerning whether cut-offs result in sufficient low false-negative and false-positives, requires the establishment of a golden standard. Deploying external multiple source data may contribute to this development.

Another limitation is the cross-sectional nature of our data. The lack of longitudinal data defers final conclusions on the cause-and-effect relationship between GASA and the criterion variables. The absence of time does not allow us to investigate the possible developmental pattern of disordered gaming.

## Conclusions

The current LCA model identifying five groups of gamers contributes to the field in terms of offering a more specific model allowing for future research to study the development of disordered gaming through these five steps.

## Author contributions

HM participated in conceptualizing and designing the study. She participated in conducting the analyses, in the interpretation of the results, and had a key role in the drafting of the article. GN participated in conceptualizing the study and had a key role in conducting the analyses and in the interpretation of the results. He drafted parts of the article, and participated in critically revising the article and approved of the final version.

### Conflict of interest statement

The authors declare that the research was conducted in the absence of any commercial or financial relationships that could be construed as a potential conflict of interest.

## Footnotes

^1^Note regarding gender. We estimated a model where gender is inserted as an active covariate to assess measurement invariance (see Hagenaars, 1990). We found that none of the bivariate residuals between gender and the GASA indicators exceeded the level of 3.84. We further assessed the invariance by not only testing whether the intercepts were different but also by allowing interaction between cluster and gender for each of the indicators. Here, it was clear that the overall fit deteriorated (BIC was higher). Hence the measure seems to be equivalent or invariant with respect to gender.

^2^Note regarding the factor structure of the GAS. In Table 1, we clearly contrasted the latent class cluster analysis with a 2-factor model with each 4 latent classes. The most appropriate model was a latent class models with 5 clusters and two correlated errors. The graphical display shows that the latent classes' curves are not intersecting This does not mean that our final model is a single factor model. It is a latent variable with unordered classes. A single factor model consists of ordered latent classes. Imposing such a constraint on the data was associated with a deterioration of fit. BIC increased to 21472,6. Hence, we cannot impose a strict unidimensional model. Allowing the residuals of the items GASA1 and GASA2 to correlate of such a single factor model also resulted in a worse fitting mode than the final model. Such a model had a BIC of 21302,6.

^3^For a more detailed view please consult the first author
